# New Tools to Study DNA Double-Strand Break Repair Pathway Choice

**DOI:** 10.1371/journal.pone.0077206

**Published:** 2013-10-14

**Authors:** Daniel Gomez-Cabello, Sonia Jimeno, María Jesús Fernández-Ávila, Pablo Huertas

**Affiliations:** 1 Centro Andaluz de Biología Molecular y Medicina Regenerativa (CABIMER), Sevilla, Spain; 2 Departamento de Genética, Universidad de Sevilla, Sevilla, Spain; National Cancer Institute, United States of America

## Abstract

A broken DNA molecule is difficult to repair, highly mutagenic, and extremely cytotoxic. Such breaks can be repaired by homology-independent or homology-directed mechanisms. Little is known about the network that controls the repair pathway choice except that a licensing step for homology-mediated repair exists, called DNA-end resection. The choice between these two repair pathways is a key event for genomic stability maintenance, and an imbalance of the ratio is directly linked with human diseases, including cancer. Here we present novel reporters to study the balance between both repair options in human cells. In these systems, a double-strand break can be alternatively repaired by homology-independent or -dependent mechanisms, leading to the accumulation of distinct fluorescent proteins. These reporters thus allow the balance between both repair pathways to be analyzed in different experimental setups. We validated the reporters by analyzing the effect of protein downregulation of the DNA end resection and non-homologous end-joining pathways. Finally, we analyzed the role of the DNA damage response on double-strand break (DSB) repair mechanism selection. Our reporters could be used in the future to understand the roles of specific factors, whole pathways, or drugs in DSB repair pathway choice, or for genome-wide screening. Moreover, our findings can be applied to increase gene-targeting efficiency, making it a beneficial tool for a broad audience in the biological sciences.

## Introduction

DNA is under the constant attack of many agents, physical and chemical, that alter its structure [1], [2]. Although those alterations are usually repaired, genomes are never completely stable [3]. This low level of genomic instability does not compromise cell or organismal survival and is the main force driving evolution [3]. However, if the cellular DNA repair pathways are mutated, genomes become increasingly unstable, a phenomena tightly related to several human pathologies, including cancer [1]–[3]. There are many types of DNA damage and, correspondingly, many DNA repair pathways [1], [2]. Many of these take advantage of the double-stranded nature of DNA to use an intact strand to recover the information lost in the damaged strand. This is not possible when a break occurs simultaneously on both strands, a so-called double-strand break (DSB). As a consequence, DSB DNA lesions are extremely difficult to repair and are highly cytotoxic and mutagenic [1]–[8]. While there are several cellular pathways that repair DSBs [4], [9], all can be grouped into one of two categories: homology-independent (non-homologous end joining; NHEJ) [6] or homology-mediated repair [8]. While the former mechanistically consists of a simple ligation of two ends, the latter is more complex and requires that a homologous sequence is used as a template for repair. There are various NHEJ repair pathways. The majority of NHEJ repairs use the classical NHEJ repair pathway, which is mediated by the DNA-PKcs-Ku70–Ku80 complex and ligase 4 [6]. However, in some circumstances DSBs are repaired by a Ku-independent repair mechanism that use microhomology as an intermediate and is dependent on ligase 3 [10], [11]; this is termed Alt-NHEJ. Similarly, there are four different homology-mediated mechanisms: the three core homologous recombination subpathways (double Holliday junction, synthesis-dependent strand annealing, and break-induced replication) and the intramolecular mechanism single-strand annealing (SSA) [9]. All of these mechanisms use a homologous molecule during the repair process, but whereas the first three require Rad51-mediated DNA invasion of the homologous partner, SSA is Rad51 independent. However, all of these are initiated by the same mechanism, a licensing step known as DNA-end resection [5]. Therefore, despite the different mechanisms and outcomes of these pathways, all can be grouped and analyzed as a single category of homology-mediated repair (HR).

DNA-end resection in human cells is controlled by the action of several proteins [5]. First, the MRN complex (Mre11-Rad50-Nbs1) recognizes the break [5]. If the break will be repaired by HR, the coordinated action of the MRN complex and CtIP protein activates a 5′-to-3′ nucleolytic degradation of both ends, close to the break (short-range resection) [5]. In a second wave (termed long-range resection), the exonuclease Exo1 and/or the helicase BLM act together with an unknown nuclease in higher eukaryotes (budding yeast BLM homologue Sgs1 acts with the nuclease Dna2), to extend the resected DNA by several kilobases [5]. Resected DNA is an essential intermediate of all homology-mediated repair pathways and also inhibits classical NHEJ due to the inability of core NHEJ proteins to bind single-stranded DNA (ssDNA) [12]. As in homology-mediated repair, Alt-NHEJ requires resected DNA to expose the microhomologies used during repair. Hence, the Alt-NHEJ pathway mechanistically shares steps with both HR and classical NHEJ.

Appropriate DSB repair is essential for cellular and organismal survival. In humans, many diseases are related to mutations in DSB repair protein-coding genes [2], [7]. However, in many cases these conditions are not caused by a specific impairment in one type of repair, but by an imbalance between homology-driven versus homology-independent repair mechanisms [9], [13]. Whereas our knowledge of the mechanisms of repair is quite extensive, how the decision between NHEJ and HR is made is still unknown. Here, we present two different systems that we specifically designed to tackle this question. We created a construct in which the formation of DSBs can be induced using the meganuclease I-SceI. In contrast to previously reported systems, which can measure either classical NHEJ or specific HR subtypes, our reporters allow us to monitor the ratio between homology-driven and homology-independent repair by emitting the distinct fluorescent signals of red for HR and green for NHEJ. We validated these reporter systems by manipulating either NHEJ or DNA-end resection. Finally, we analyzed the effect of downregulating different DNA damage checkpoint proteins on the balance between these repair pathways.

## Materials and Methods

### Cloning of SSR Systems

**Figure 1:**
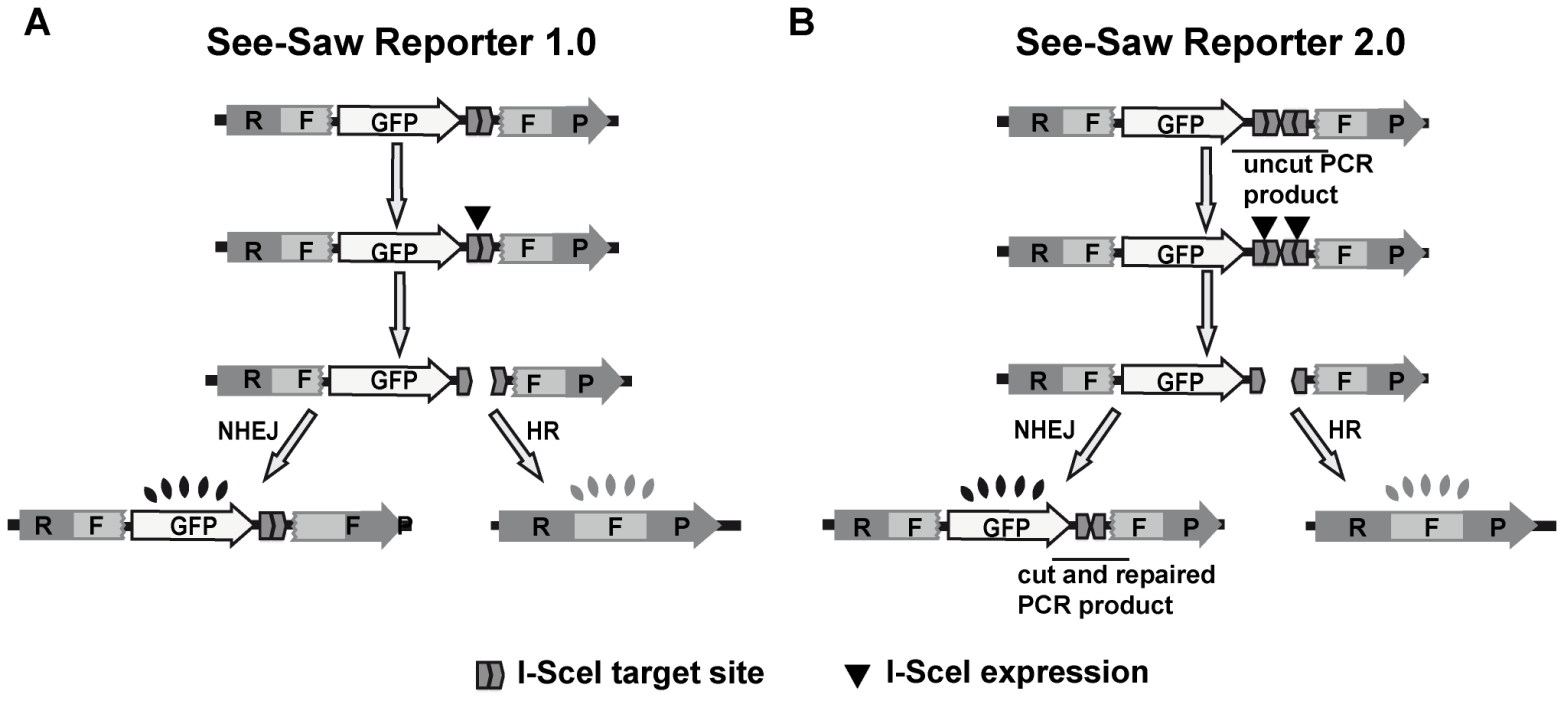
Graphical representation and experimental validation of the HRNH1.0 reporter. Schematic representation of the SeeSaw reporter (SSR) 1.0 (A) and SSR 2.0 (B). A GFP gene is flanked by two truncated parts of the RFP gene (RF and FP) that share 302 bp of homologous sequence. One I-SceI target site in SSR 1.0 (A), and two in opposite orientation in SSR 2.0 (B), were cloned at the 3′ end of the *GFP* gene. Expression of I-SceI generates a DSB; if the damage is resolved by NHEJ, cells will express the GFP protein, while if it is repaired using homologous sequence by HR, cells express the *RFP* gene. The PCR products used in Figure 2F are depicted as “uncut PCR fragment” and “cut and repaired PCR fragment”.

Both SSRs (Figure 1A and 1B) were derived from the pEGFP-C1 plasmid. For nuclease-cleavage-sequence cloning, two annealed oligonucleotides bearing either one (SSR 1.0; 5′-AATTCAGTTACGCTAGGGATAACAGGGTAATATAGtaaaatCTATATTACCCTGTTATCCCTAGCGTAACT-3′) or two (SSR 2.0; 5′-AATTCACTAGGGGATAACAGGGTAATAATAATTACCCTGTTATCCCTATG-3′ and 5′-AATTCATAGGGATAACAGGGTAATTATTATTACCCTGTTATCCCTAGTG-3′) inverted I-SceI target sequences were inserted at the 3′ end of the *GFP* gene. Both *RFP* repeats were obtained from the Hc-Red plasmid by PCR. An *Age*I-*Age*I fragment harboring the 5′ end of the gene (RF) was obtained using the oligonucleotides 5′-ATGTCGTAACAACTCCGCC-3′ and 5′-GGACTTACCGGTCCGCTCTTGTTCTTCATC-3′ and cloned at an *Age*I restriction site located between the CMV promoter and the *GFP* gene, maintaining the reading frame. A *Sal*I-*BamH*I PCR product bearing the 3′ end of the *RFP* gene (FP) was obtained using the oligonucleotides 5′-GGACTTGTCGACACCCAGAGCATGAGAATCCAC-3′ and 5′-GTCGACGGATCCTGCAGAATTCGAAGCTTGAGCTCGAGA-3′ and cloned at *Sal*I and *BamH*I sites, just behind the I-SceI site(s). The RF and FP fragments share 302 bp of homology (Figure 1A and 1B). The original SSR 1.0 reporter showed low GFP expression. To boost it, we inserted a PCR fragment containing the woodchuck hepatitis post-transcriptional regulatory element (WPRE) at *Kpn*I-*Sal*I restriction site to stabilize the mRNA.

### Cell Culture and Drug Treatments

Both SSR reporters were integrated in U2OS osteosarcoma cells (ATCC #HTB-96) by plasmid transfection using Fugene6 Transfection Reagent (Roche) according to the manufactureŕs instructions. Cells were cultured in high-glucose Dulbecco’s Modified Eagle Medium (DMEM) supplemented with 10% FBS, 2 mM Glutamine, 100 µg/ml streptomycin, 100 U/ml penicillin, and 0.5 mg/ml G418 at 37°C in 5% CO_2_. U2OS stably expressing SSR systems were selected with 0.5 mg/ml G418 antibiotics. Single clones of both reporter systems were obtained by plating 1000 cells in 15 cm diameter plates and selection of isolated colonies.

### Southern Blot

Southern blot analyses were performed according to standard procedures with ^32^P-radiolabelled probes. The DNA probe of a *GFP* fragment obtained by PCR amplification (using the primers 5′-CACGAACTCCAGCAGGACCATG-3′ and 5′-CTGGTCGAGCTGGACGGCGACG-3′) was labeled with ^32^P. The product was labeled with 500 µg/ml N6 random primers, 0.03 mM each of dATP, dGTP, and dTTP, 0.01 dCTP, 1 mCi/ml [^32^P]dCTP, and Klenow polymerase during 2 hr at 37°C. The labeled product was purified with G25 columns (Amersham, NAP5). To obtain the DNA cells were growth in 10 cm plates to confluence, collected by trypsinization, and resuspended in 300 µl of lysis buffer (50 mM Tris pH 7.5, 100 mM NaCl, 0.5% SDS, and 5 mM EDTA) with 20 µg/ml proteinase K, and incubated overnight at 55°C with agitation. DNA was precipitated with 1 volume of isopropanol and centrifuged at 13000 g. The DNA pellet was washed with 70% ethanol and resuspended in 40 µl H_2_O. DNA (40 µg) was digested with *EcoR*I overnight at 37°C, resolved on a 0.8% agarose gel, and transferred to a Hybond N+membrane (Amersham) by capillarity blotting. The membrane was hybridized with the GFP-labeled fragment in hybridization buffer (0.25 M Na_2_HPO_4_, 0.2% H_3_PO_4_, 7% SDS, and 1 mM EDTA) overnight at 65°C, washed three times at 50°C with washing buffer (0.1% SSC, 1% SDS), and quantified in a FUJI FLA5000.

### Genomic DNA PCR

Genomic DNA was extracted from U2OS cells stably transfected with the SSR2.0 system, as indicated in the Southern blot section. Samples were collected 24, 48, and 72 hr after I-SceI lentiviral transduction. PCR was performed using primers flanking the I-SceI target site (Fw 5′-CACGAACTCCAGCAGGACCATG-3′ and Rv 5′-ATGTTTCAGGTTCAGGGGGAGG-3′) or inside the actin gene for 30 amplification cycles. PCR products were resolved in 4% native acrylamide/bisacrylamide gel (I-SceI target site) or 1.5% agarose (actin PCR), stained with RedSafe (Intron Biotechnology, ref 21141) for 10 min, visualized with UV light, and documented using a Gel-Doc XR+System (BioRad). The resultant image was quantified using the Quantity One software (BioRad). The ratio between the density of the band from the reporter and the one from the actin gene was normalized to the time 0 (e.g. before I-SceI infection).

### Lentiviral Production and Infection

Lentiviral particles harboring the *I-SceI* gene were generated using 10 µg p8.91, 5 µg pVSV-G, and 15 µg pRRL_sEF1a_HA.NLS.SceOPT.T2A.TagBFP vectors (Andrew Scharenberg) by calcium phosphate transfection in A293T cells. After 48 hr, lentiviruses were collected from the media by 100,000 g centrifugation for 2 hr at 4°C. The virus titer was calculated by infecting U2OS cells with fixed amounts of the virus suspension and analyzing the percentage of BFP-expressing cells by FACS.

Lentiviral particles bearing short hairpin RNA (shRNA) against different messenger RNAs (see Table S1 for the list and access numbers) were obtained from Sigma Aldrich. Infection was performed according the manufactureŕs manual. U2OS cells stably expressing the shRNAs were selected by adding 1 µg/ml puromycin to the medium after infection.

### Flow Cytometry

Inhibitor-treated or shRNA-depleted samples were prepared for FACS analysis as following. Cells were seeded in 12-well plate (50,000 cells per well) in duplicate. The next day, cells were infected with lentivirus particles containing I-SceI–BFP expression construct at MOI 5 using 8 µg/ml polybrene in 300 µl DMEM. After 6 hr, media was exchanged with fresh DMEM. For inhibitor-treated samples, drugs or, as a control, the vehicle DMSO was added at this point. After 16–18 hr, cells were washed with PBS, trypsinized, neutralized in DMEM, and centrifugated at 800 g. Cells were resuspended, fixed with 4% paraformaldehyde for 20 min, and collected by centrifugation. Pellets were then washed twice with PBS and resuspended in 200 µl PBS. Samples were analyzed with a BD FACSAria with the BD FACSDiva Software v5.0.3. Five different parameters were considered: side scatter (SSC), forward scatter (FSC), blue fluorescence (407 nm violet laser BP, Filter 450/40), green fluorescence (488 nm blue laser BP Filter 530/30), and red fluorescence (488 nm blue laser BP Filter 575/26). The number of green and red cells from 10,000 events positives for blue fluorescence (infected with the I-SceI–BFP construct) was measured. The average of both duplicates was calculated for each experiment. At least two completely independent experiments were carried out for each condition.

### Microscopy

About 4000 U2OS cells stably transfected with the SSR system bearing different shRNAs were seeded. I-SceI-BFP Lentiviral transduction were performed using an MOI 5, changing the media after 24 hr. Cells were grown during 48 hr, fixed with 4% paraformaldehyde, and washed with PBS prior visualization with a fluorescent microscope.

### Homology-dependent and Homology-independent Ratio Quantification

The HR/NHEJ ratio was calculated by dividing the number of cells expressing GFP from the subpopulations expressing BFP by the number of cells expressing RFP from the subpopulations expressing BFP. To avoid noise due to the random events that create RFP-expressing cells prior to I-SceI induction, a parallel experiment without I-SceI infection was performed for each shRNA-infected cell line and used as background. The HR/NHEJ ratio was normalized to the cells expressing the shRNA control.

## Results and Discussion

### A Novel Set of Reporters to Study the Balance between Homology Dependent and Independent Repair

As DNA resection dictates the way a DSB is going to be repaired [5], [9], we have designed a resection-sensitive set of reporters to measure the balance between NHEJ and HR. The goal was to detect changes in the ratio between repair pathways, irrespective if they were caused by increase and/or decrease of HR or NHEJ. We called this constructs the SeeSaw Reporters (SSR), as a deviation towards any directions does not depend on one repair mechanism but rather on the combination of both. Hence, a net bias towards HR or NHEJ in our reporters does not necessarily reflect an increase or decrease of either one but rather an imbalance between both (Figure 1).

The SSR consists of the *GFP* gene flanked by a 3′- and a 5′-end truncated portion of the *RFP* gene that share 302 bp of homology with each other (Figure 1a and 1b). The sequence recognized by the meganuclease I-SceI was inserted at the 3′ end of the *GFP* gene. It is worth noting that the reporters were designed to express the *GFP* gene constitutively. We decided to use this approach rather than the conventional approach, which is to place the I-SceI at the beginning of the gene and disrupt the reading frame, so that cells with the reporters do not express any fluorescent signal prior to repair. Our rationale was that the conventional way would allow us to observe only those NHEJ events in which mutagenic repair restored the reading frame. In other words, conventional reporters do not detect classical error-free NHEJ or the majority of mutagenic end-joining (e.g. those events that do not restore the appropriate reading frame). In contrast, our reporters allow us to observe both error-free and error-prone NHEJ.

**Figure 2:**
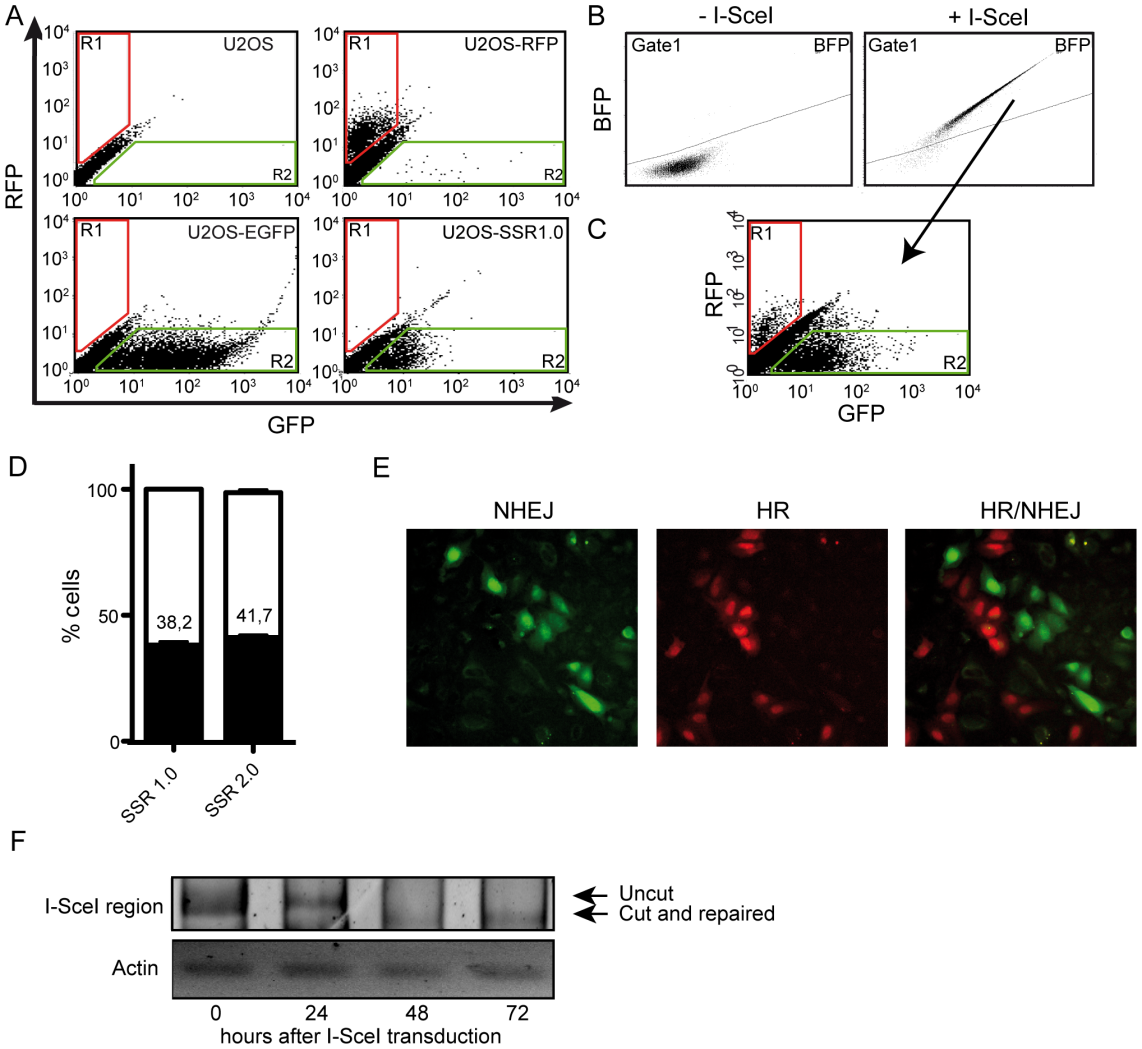
Initial characterization of the SSR systems. (A) Flow cytometer analysis of U2OS osteosarcoma cells transfected or not with control vectors, pEGFP and pHc-RFP, or the SSR 1.0 system. For quantification, two regions (R1 and R2) were established following the comparison of U2OS cells and cells transfected with control plasmids. R1 encompasses cells that fluoresce red above background levels, whereas R2 encompasses cells that fluoresce green above background levels. (B) Cells stably expressing the SSR 1.0 and an shRNA against a scrambled sequence were transfected or not with the I-SceI–BFP construct and analyzed for the presence of blue fluorescence. A gated region (gate1) was created to separate cells that expressed the BFP protein. (C) Cells from Figure 1D that fell into the gate1 region were analyzed for red or green fluorescence, using R1 and R2 as defined in Figure 2A. (D) Quantification by flow cytometry of the percentage of cells expressing GFP or RFP after I-SceI–BFP lentiviral infection in cells harboring the SSR 1.0 or SSR 2.0 systems and a scrambled shRNA. Percentages were calculated as R1 (black, HR) or R2 (white, NHEJ) versus cells that expressed any fluorescence (R1+R2). Data represent a minimum of three sets of duplicated experiments. Average percentage and standard error of HR events is shown. (E) A sample image under a fluorescent microscope of cells harboring the SSR 1.0 system of cells that were repaired by NHEJ (green) or HR (red). (F) PCR analysis of the cleavage efficiency and repair by NHEJ in the SSR 2.0 system. PCR products were obtained with oligonucleotides located at the end of the GFP gene and the beginning of the FP fragment (see Figure 1B). Cleavage with I-SceI impaired the PCR reaction, resulting in a reduction of the uncut fragment (see also Figure 1). Upon NHEJ repair, a new, faster migrating species appeared (cut-and-repaired fragment; see Figure 1B). A PCR product of the actin gene using the same genomic DNA was used as loading control.

We designed two different reporters: SSR 1.0 with a single I-SceI target site (Figure 1A) and SSR 2.0 with two I-SceI target site (Figure 1B). In the single-site reporter, I-SceI cleavage generates ligatable, 3′-overhanging ends that can be joined accurately by NHEJ to re-create the I-SceI target site, which can in turn be cut again by the enzyme. This cycle can be repeated endlessly until the I-SceI site disappears either by homology-mediated repair or mutagenic end-joining. Thus, one caveat this system shares with previously published reporters is that it is biased to mutagenic repair. To study the extent of this bias in our reporter, we generated the version with two I-SceI sites, which are close to each other in an inverted orientation (Figure 1B). The I-SceI target site is not palindromic, and hence the repair of two inverted I-SceI–mediated breaks by NHEJ always destroys the target sequence and the intervening sequence if they occur simultaneously. Indeed, when we analyzed the break repair of SSR 2.0 by PCR, we only observed the cut-and-repair band corresponding to a double-cleavage event (Figure 2F). Thus, in this new See-Saw Reporter 2.0, both NHEJ and HR destroy the I-SceI site, and the majority of the repair events represent a single cycle of breakage-repair.

Infection with a lentivirus harboring the I-SceI gene creates DSBs. When cells repair such breaks through a classical NHEJ-type of repair, the *GFP* gene is restored and the cells fluoresce green (Figure 1A, 1B, 2A–2E). When resection takes place, thereby inhibiting classical NHEJ, the homologous regions of the *RFP* gene are exposed and used to repair the break by SSA [5], [8], [9]. In this case, the repair creates a functional *RFP* gene and eliminates the *GFP* gene, and the cells fluoresce red (Figure 1A, 1B, 2A–2E). One additional possibility is that resection takes place but the repair is mediated by alt-NHEJ. In this case, cells might lose the intervening region, e.g. the *GFP* gene, without creating a functional *RFP* gene [4], [9], [10]. In this case, cells would fluoresce neither green nor red and should thus be invisible in our system. Unfortunately, in all our stably transfected cells, there were always a significant percentage of cells that fit this condition (see tables S2–S7). We reasoned that these cells represent an heterogenous population formed by: *i*) cells that have not yet repaired the break, or that have repaired it too recently for the fluorescent protein to accumulate to large enough levels to be visualized by FACS; *ii*) cells that maintain resistance but have lost the reporter and thus express neither GFP nor RFP; or *iii*) cells that have repaired the break in a way that hamper both *RFP* and *GFP* expression, such as alt-NHEJ.

One possible caveat of this approach is that those cells in which I-SceI is not present will remain green, affecting the measurement of NHEJ events. To analyze only cells that were infected with I-SceI, and therefore in which a DSB had been induced, we used a blue-fluorescent protein (BFP)–I-SceI construct (Figure 2B) and restricted the analysis to those cells that fluoresce blue. Also, cells in which I-SceI was present but in which the DNA had not been cleaved continued to express GFP. However, as can be seen in Figure 2F for SSR 2.0, the percentage of cells in which this happened was negligible: 24 hr after infection, 40% of the cells with the reporter were uncut, while after 48 hr, less than 1% of the report was uncut. In addition, at 48 hr, a smaller band corresponding to the joined molecule that had lost one copy of the I-SceI site as well as the intervening sequence began to accumulate (Figure 2F). It can be argued that any GFP protein produced before I-SceI cleavage would still be present in the cells. However, if this were true, RFP-positive cells would also be positive for GFP expression, at least in some cases. As can be seen in Figure 2E, we never observed such cells, implying that GFP expression measured in our reporters corresponded to GFP that was expressed after DNA repair had occurred.

Therefore, these reporters allow us to analyze the balance between homology-dependent and -independent repair by determining the percentage of green versus red cells in the population by FACS (Figures 2A–2D) or microscopy (Figure 2E). We decided to use a SSA reporter as an indicator of homology-mediated repair, rather than a classical recombination reporter, mainly due to its increased efficiency. Rad51-mediated recombination is a rare event, and we reasoned that it would be difficult to observe changes when compared with NHEJ. To analyze the effects of different cellular processes in the balance between DSB repair pathways, we stably integrated the reporter into U2OS cells. For our studies, we used clones that harbored single-copy integration of the reporters (Figure S1).

In a population of U2OS cells stably transfected with the SSR reporters, we observed cells that expressed green fluorescence above background levels even without I-SceI transfection (compare U2OS with U2OS-SSR 1.0 cells) Figure 2A; region 2, R2) These depicts cells with the SSR that constitutively expressed GFP. We also observed a low number of cells (less than 1%) that emitted red fluorescence (Figure 2A; region 1, R1), which we interpreted to be spontaneous SSA events that were not triggered by I-SceI cleavage. As mentioned above, we only analyzed cells that harbored the I-SceI-BFP construct (Figure 1D). Two days after I-SceI infection, we prepare the cells as described in the Methods section and analyzed them by FACS (Figure 2B and 2C) or microscopy (Figure 2E) for blue, green, and red fluorescence. Continuous expression of I-SceI for two days led to almost 100% cleavage of the reporter (Figure 2F). To calculate the balance between HR and NHEJ, we considered the cells that expressed the I-SceI–BFP construct (Figure 2B, gate 1). Those cells that fall into Gate1 are then analyzed for the appearance of red (in R1) or green fluorescence (in R2) (Figure 2C). We calculated the ratio between homology-mediated versus homology-independent repair (R1/R2) (Figure 2D). Similar results were obtained with the SSR 1.0 and SSR 2.0 reporters, indicating that repair balance is not strongly biased in any of the systems (Figure 2D) in control cells. We observed that for both reporters, and as measured both by FACS or microscopy, cells showed a 4∶6 ratio of HR compared to NHEJ; i.e., 40% of the cells repair using the homologous sequence. Although this proportion might seem high for homology-mediated repair, it is worth pointing out that SSA is an extremely efficient pathway when compared to Rad51-mediated recombination [14], [15].

**Figure 3:**
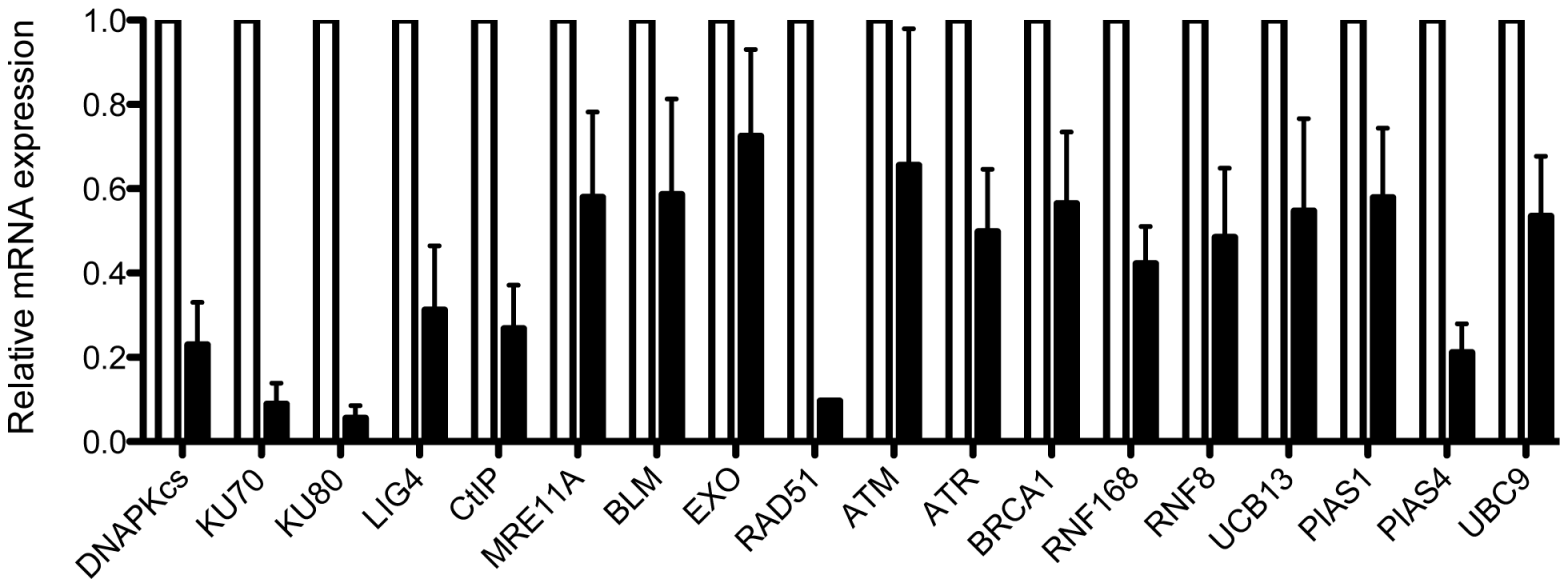
Reduction of the RNA levels upon shRNA-mediated depletion against several target genes. Quantitative RT-PCR data was measured for mRNA in cells stably transfected with the indicated shRNAs. The mRNA level of each gene was normalized to actin mRNA. The mRNA level of each gene in each cell line (black bars) was normalized to the levels in a cell line stably transfected with an shRNA with a scrambled sequence (white bars). Average and s.e.m. of a minimum of three experiments with triplicates is plotted.

**Figure 4:**
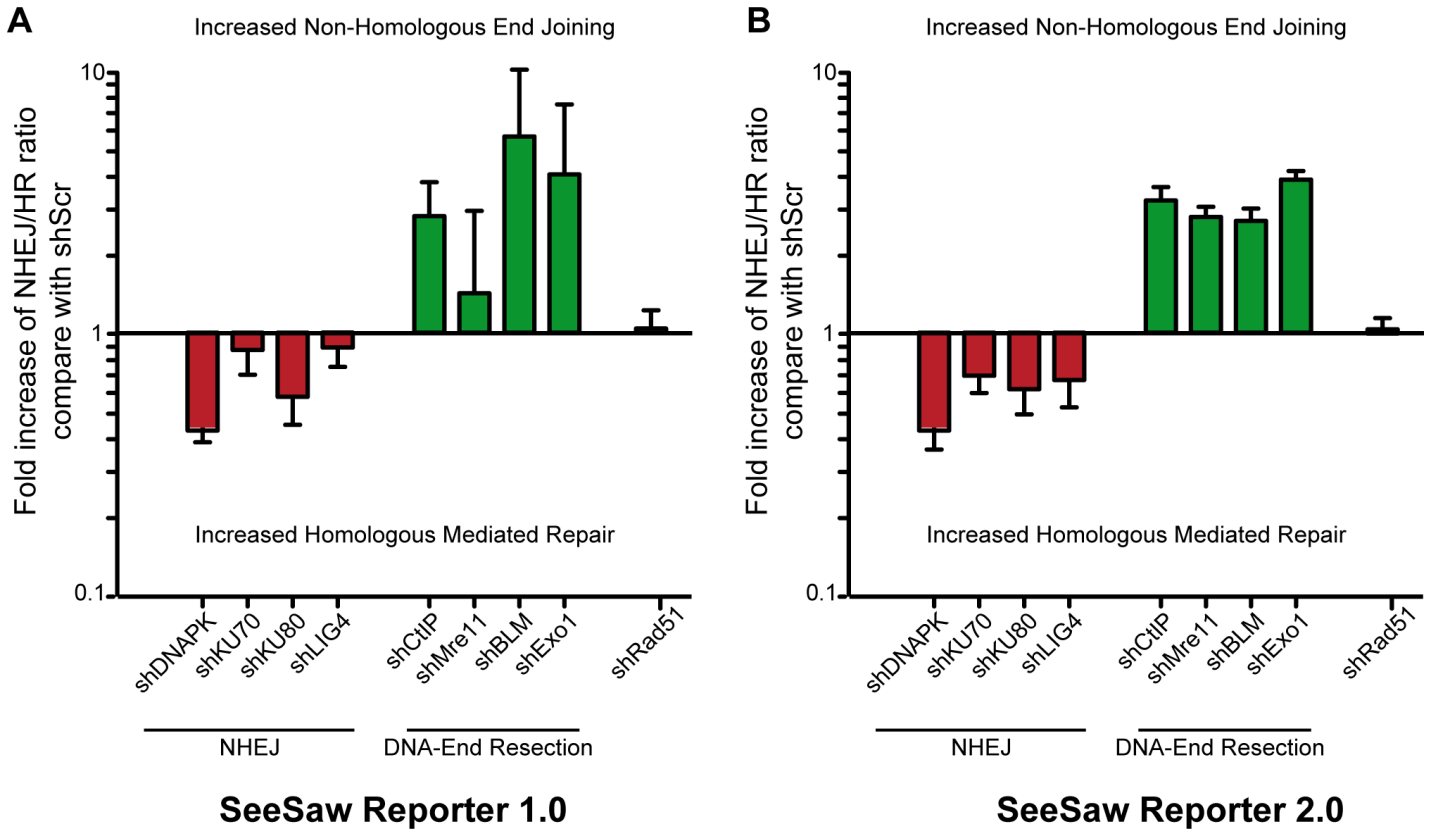
Sensitivity of SSR systems to NHEJ and DNA send resection impairment. Effects of shRNA-mediated depletion of core NHEJ or DNA end resection factors for the SSR 1.0 (A) and SSR 2.0 (B). To calculate the deviation from the balance between homology-dependent versus homology-independent repair, the ratio between green versus red cells in each conditions was calculated. To facilitate comparing experiments, this ratio was normalized for each shRNA with a scrambled sequence shRNA as a control. Those shRNAs that skewed the balance towards an increase in homology-independent repair have a fold-increase of over 1 (green bars), while those with an increase in HR have a fold-decrease of less than one (red bars). Data represent a minimum of three sets of duplicated experiments.

As a proof-of-concept for the SeeSaw reporters, we next analyzed the impact of blocking established NHEJ and resection factors in repair balance. [6]. The extent of mRNA depletion obtained by shRNAs used in this work is shown in Figure 3. To facilitate comparison between different reporters and experiments, we normalized the HR/NHEJ ratio to the scramble control, and plotted the fold increase values side-by-side (see Methods for details; Figure 4). Any values greater than 1 represent a preference for homology-independent repair; values less than 1 mean a bias towards homology-dependent repair.

### Hindering NHEJ Drives Cells to Repair Via a Homology-driven Repair

It has been previously reported that impairing NHEJ drives cells to increase DNA-end resection and homologous recombination in several organisms [5], [16]–[20]. Thus, we downregulated several key players of NHEJ, namely, the three subunits of the DNA-PK complex (DNA-PKcs, Ku70, and Ku80) and ligase 4 (Lig4). As expected, we observed a deviation of the balance towards an increase of homology-mediated repair (Figure 4, Tables S2 and S3). These results agree with previously published reports that indicate that, in the absence of NHEJ, DNA-end are unprotected, resection occurs, and the breaks are more prone to be repaired by homologous recombination [5], [16]–[21]. Although we observed a similar trend for both SSR 1.0 and 2.0, it was clearer in the latter (Figure 4). We believe this is due to the fact that accurate NHEJ in the SSR 1.0 system renders a functional I-SceI target site that can be recleaved. Thus, since only one cycle of break-repair takes place for SSR 2.0, the full extent of the role of NHEJ, including accurate and mutagenic, is discernable, whereas error-free repair renders a re-cleavable target site and favors mutagenic repair for SSR 1.0, making the picture less clear.

### DNA end Resection Controls the Balance between Homology-dependent and -Independent Repair

DNA end resection is the main process controlling the choice between DSB repair pathways by licensing HR and inhibiting NHEJ [4], [5], [9]. To validate our reporters, we analyzed the effect of hindering resection by protein depletion (Figure 4, Tables S4 and S5). shRNA-mediated depletion of cells for CtIP, BLM, or Exo1 strongly skewed our reporter towards NHEJ rather than HR, as compared to control cells with a scrambled shRNA sequence. When we compared the HR/NHEJ ratio, normalized to that from the scrambled shRNA control, depletion of CtIP, BLM and Exo1 altered the ratio towards an increase in NHEJ (Figure 4). Therefore, this reporter responded strongly to both short-range and long-range DNA end resection. Mre11 has an essential role in short-range resection but has also been implicated in other processes, such as NHEJ, tethering both sides of the break, and DSB sensing and checkpoint activation [22]. Thus, the outcome of Mre11 depletion on DSB repair pathway choice is difficult to predict and highly complex, since it would negatively affect both NHEJ and HR. This is the type of question our reporters were specifically designed to answer. When we depleted Mre11 in our cells, we observed a deviation of the balance towards NHEJ, demonstrating that Mre11 strongly favors homology-driven repair. (Figure 4). This effect is likely due to the role Mre11 plays in DNA end resection [22].

Thus, we confirmed that hindering DNA end resection clearly skews the balance towards NHEJ. Similar effects were observed after impairment of short-end and long-end resection (Figure 4). Importantly, although we did not observe qualitative differences between SSR 1.0 and SSR 2.0, we clearly distinguished quantitative differences.

**Figure 5:**
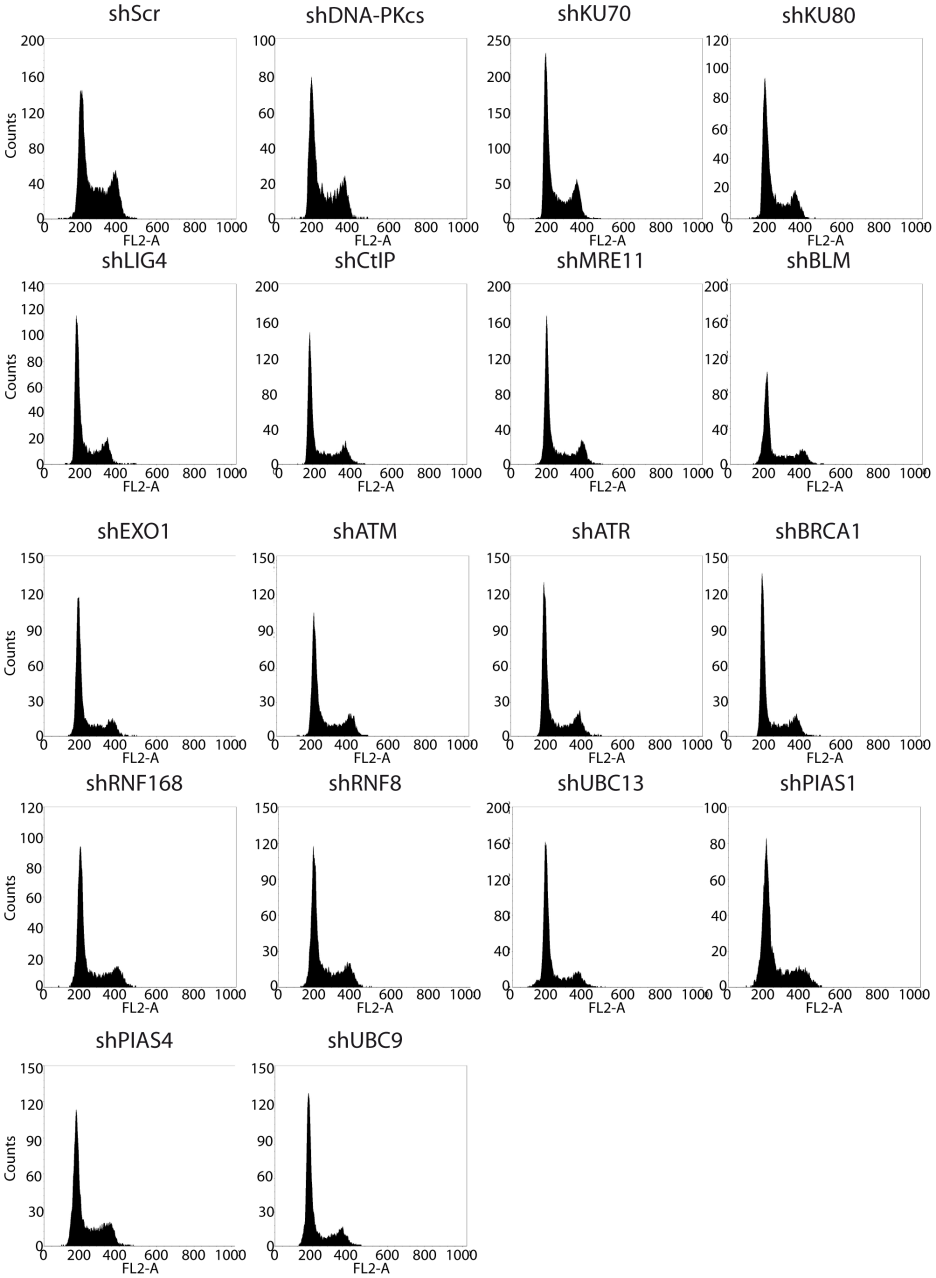
Cell cycle distribution after shRNA-mediated depletion of several target genes. A representative FLOW cytometry plot after downregulation of the indicated target genes with shRNA is shown.

To confirm that the data obtained so far reflect the role of DNA end resection and NHEJ in DSB repair pathway choice, and that they were not due to spurious effects, we performed a rigorous series of controls. First, it has been demonstrated that the cell cycle is the major regulator of the election between homology-independent and homology-dependent repair [5], [23]–[26]. Whereas G1 cells can only use NHEJ, S and G2 cells can activate HR through a CDK-mediated licensing of DNA end resection [5], [23]–[26]. We therefore analyzed cell cycle profiles of cells depleted for all the factors used in this study; we observed no differences that could explain the imbalance in repair pathway choice (Figure 5). As an additional control, we decided to deplete a protein related to DSB repair that should have no effect on repair pathway choice, namely, Rad51. Rad51 is involved in late steps of homologous recombination, following the decision for HR or NHEJ [4], [9], [15]. More importantly, Rad51 is not involved in SSA [4], [9], [15], hence avoiding indirect effects due to the repair mechanism itself rather than pathway choice. Indeed, depletion of Rad51 had no effect on the SeeSaw reporters (Figure 4).

### The DNA Damage Response and the Regulation of DSB Repair Pathway Choice

The cellular context determines which is the best way to repair a DNA break. The coordination between DNA repair and cellular metabolism relies on a complex signal transduction cascade known as DNA damage response (DDR) [1], [2]. We decided to investigate the role of different DDR factors on influencing the balance between HR and NHEJ.

**Figure 6:**
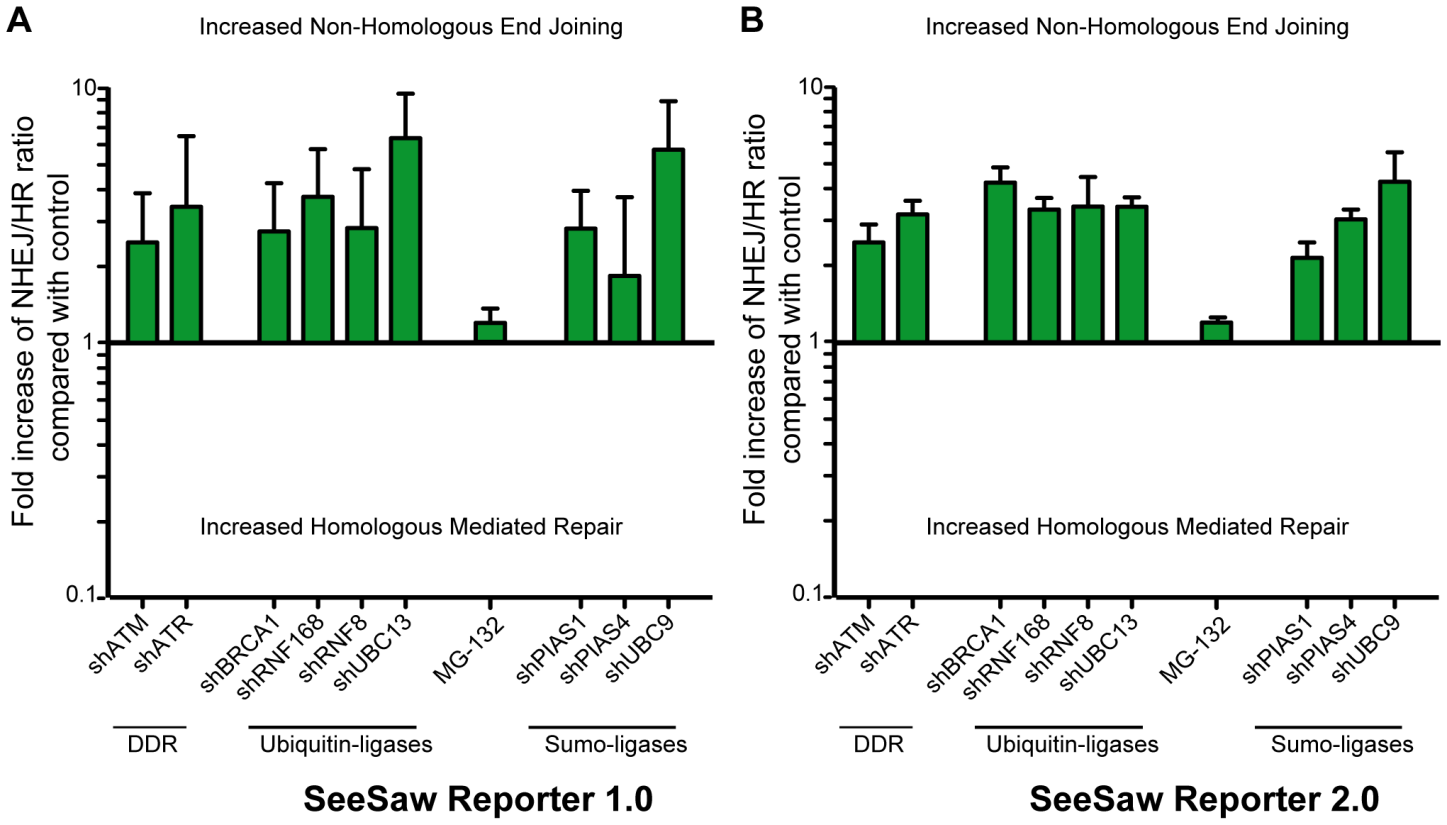
DDR defects lead to an imbalance of the HR/NHEJ ratio. The HR and NHEJ balance in the SSR 1.0 (A) and SSR 2.0 (B) in cells depleted of the checkpoint factors ATM, ATR, BRCA1, RNF169, RNF8, UBC13, UBC9, PIAS1, or PIAS4, or after MG-132 inhibition of proteasome activity. The details are as given in Figure 4, except that data for the MG-132 results were normalized to cells treated with DMSO as a control.

DDR is initiated by a series of protein phosphorylation by two kinases, ATM and ATR [1], [2]. shRNA-mediated depletion of either ATM or ATR resulted in a pronounced swing of the homology-mediated versus -independent repair balance, towards the latter (Figure 6). These results agree with a role of both ATM and ATR in favoring DNA end resection. DDR-mediated phosphorylation triggers the recruitment of multiple proteins to the vicinity of the breaks, including the E3-ubiquitin ligases RNF8, RNF168 and BRCA1. The activities of all three of these ligases are essential for DDR and DNA repair. Here, we showed that downregulation of RNF8, RNF168, or BRCA1 also skewed the balance towards NHEJ (Figure 6). This effect was specific for DNA damage–dependent ubiquitination, as similar (but more pronounced) results were obtained upon downregulation of the DNA damage–related E2 ubiquitin ligase UBC13 [27] (Figure 6), but general impairment of protein ubiquitination with the proteasome inhibitor MG132, which causes ubiquitin depletion, did not significantly alter the HR/NHEJ balance (Figure 6). Additionally, sumoylation takes place at the sites of breaks due to the activity of the E2-sumo ligase UBC9 and the E3-sumo ligases PIAS1 and PIAS4 [28]. shRNA-mediated depletion of any of these also imbalanced the HR/NHEJ ratio towards NHEJ (Figure 6). Therefore, we conclude that DDR-mediated phosphorylation, ubiquitylation, and sumoylation are involved in the DSB pathway choice and are required for HR.

## Conclusions

In summary, we have designed specific reporters to study the balance between homology-directed and homology-independent repair of DSBs. These systems can be used to analyze in an unbiased way the effect of any factor on this repair pathway choice. This will allow us to isolate and characterize new factors involved in this regulation. For our reporters, it is irrelevant if a factor has an increased or reduced ability to perform either HR or NHEJ or both, since we instead study its role in maintaining the balance. Our systems could be applied to understand one specific factor or an entire pathway, or to genome-wide screenings and drug discovery. Moreover, our findings can be applied to increase gene-targeting efficiency, a beneficial tool for a broad audience in the biological sciences.

## Supporting Information

Figure S1 **Single-integration clones of the HRNH1.0 reporter.** A, Southern blot analysis of HRNH1.0 integration in selected clones. Red arrows show clones harboring a single copy of the reporter. B, Flow cytometer analysis of single copy clones to determine the basal expression of GFP and RFP fluorescence depending of the integration site.
(TIF)

Table S1 **List of shRNA used in this work.**
(DOCX)

Table S2 **Percentage of GFP and RFP expressing-cells from the BFP-positive pool in the SSR 1.0 system upon shRNA-mediated downregulation of NHEJ factors.**
(DOCX)

Table S3 **Percentage of GFP and RFP expressing-cells from the BFP-positive pool in the SSR 2.0 system upon shRNA-mediated downregulation of NHEJ factors.**
(DOCX)

Table S4 **Percentage of GFP and RFP expressing-cells from the BFP-positive pool in the SSR 1.0 system upon shRNA-mediated downregulation of DNA resection.**
(DOCX)

Table S5 **Percentage of GFP and RFP expressing-cells from the BFP-positive pool in the SSR 2.0 system upon shRNA-mediated downregulation of DNA resection.**
(DOCX)

Table S6 **Percentage of GFP and RFP expressing-cells from the BFP-positive pool in the SSR 1.0 system upon shRNA-mediated downregulation of the DNA damage response.**
(DOCX)

Table S7 **Percentage of GFP and RFP expressing-cells from the BFP-positive pool in the SSR 2.0 system upon shRNA-mediated downregulation of the DNA damage response.**
(DOCX)
