# Supplementary figures and images for: New Tools to Study DNA Double-Strand Break Repair Pathway Choice
Source: PLoS One. 2013 Oct 14;8(10):e77206. doi: 10.1371/journal.pone.0077206 (PMC3796453; doi:10.1371/journal.pone.0077206)

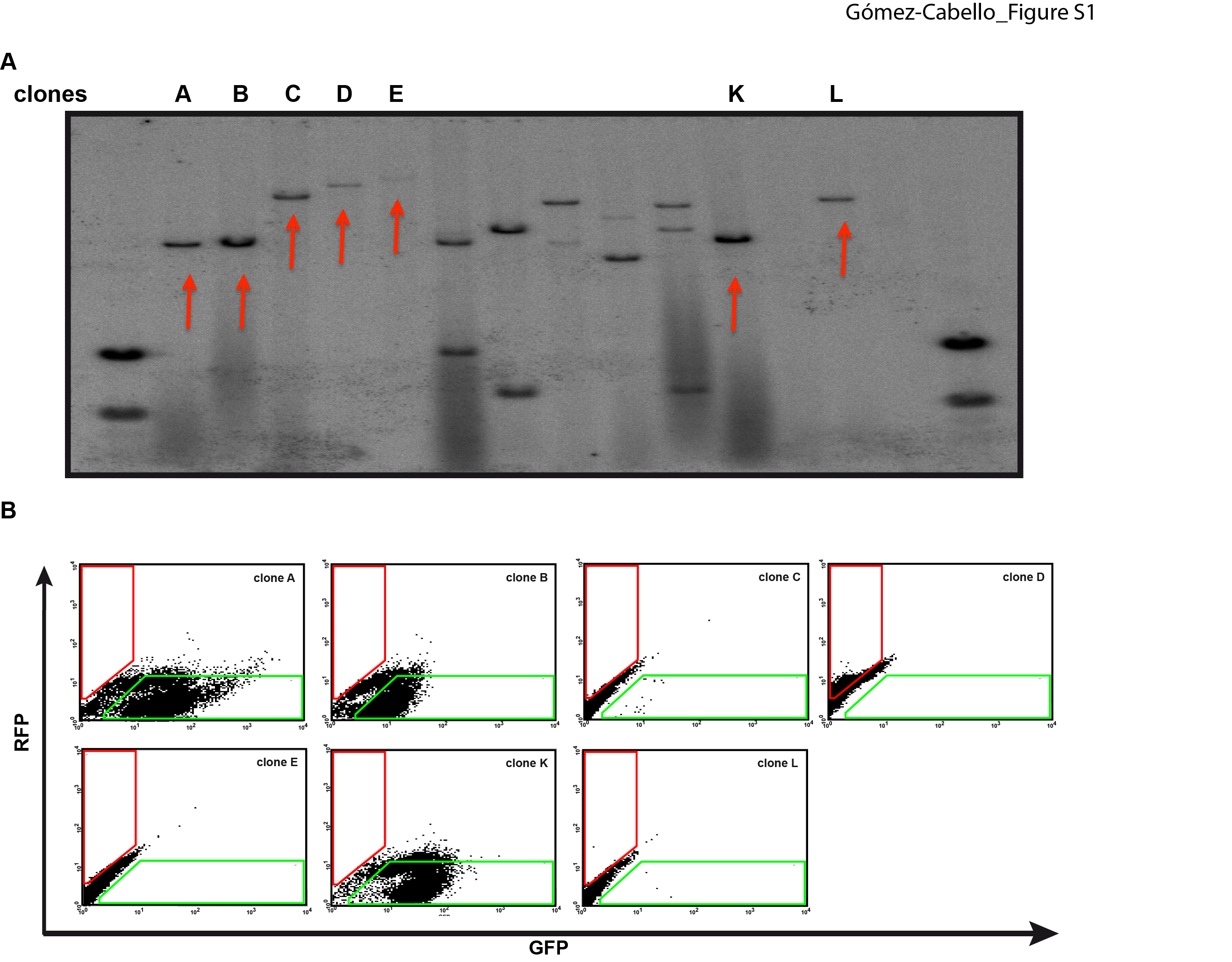

Supplement: Figure S1 — Single-integration clones of the HRNH1.0 reporter. A, Southern blot analysis of HRNH1.0 integration in selected clones. Red arrows show clones harboring a single copy of the reporter. B, Flow cytometer analysis of single copy clones to determine the basal expression of GFP and RFP fluorescence depending of the integration site. (TIF) [file pone.0077206.s001.tif]
