# Supplementary material for: New Tools to Study DNA Double-Strand Break Repair Pathway Choice
Source: PLoS One. 2013 Oct 14;8(10):e77206. doi: 10.1371/journal.pone.0077206 (PMC3796453; doi:10.1371/journal.pone.0077206)
Supplement: Table S1 — List of shRNA used in this work. (DOCX) [file pone.0077206.s002.docx]

**Table S1**

| Gene | TRC number |
| --- | --- |
| DNAPKcs | TRCN0000006255 |
| Ku70 | TRCN0000009846 |
| Ku80 | TRCN0000010467 |
| LIG4 | TRCN0000009847 |
| RBBP8 (CtIP) | TRCN0000005403 |
| MRE11A | TRCN0000039872 |
| BLM | TRCN0000004906 |
| EXO1 | TRCN0000010331 |
| ATM | TRCN0000010299 |
| ATR | TRCN0000010300 |
| BRCA1 | TRCN0000009823 |
| PIAS1 | TRCN0000010853 |
| PIAS4 | TRCN0000004118 |
| RNF168 | TRCN0000034136 |
| RNF8 | TRCN0000003438 |
| UBC13 | TRCN0000007213 |
| UBC9 | TRCN0000320374 |
