# Supplementary material for: New Tools to Study DNA Double-Strand Break Repair Pathway Choice
Source: PLoS One. 2013 Oct 14;8(10):e77206. doi: 10.1371/journal.pone.0077206 (PMC3796453; doi:10.1371/journal.pone.0077206)
Supplement: Table S2 — Percentage of GFP and RFP expressing-cells from the BFP-positive pool in the SSR 1.0 system upon shRNA-mediated downregulation of NHEJ factors. (DOCX) [file pone.0077206.s003.docx]

**Table S2: Percentage of GFP and RFP expressing-cells from the BFP-positive pool in the SSR 1.0 system upon shRNA-mediated downregulation of NHEJ factors**

| shRNA | % RFP positive cells | | % GFP positive cells | | % GFP and RFP negative cells | |
| --- | --- | --- | --- | --- | --- | --- |
|  | **Average** | **SD** | **Average** | **SD** | **Average** | **SD** |
| Scramble | 9,00 | 0,71 | 14,06 | 1,10 | 76,94 | 1,81 |
| DNAPK | 14,65 | 0,25 | 9,71 | 0,95 | 75,64 | 1,20 |
| KU70 | 11,05 | 2,09 | 17,06 | 6,08 | 71,89 | 8,16 |
| KU80 | 10,50 | 0,35 | 9,82 | 1,58 | 79,68 | 1,23 |
| LIG4 | 9,50 | 0,35 | 13,43 | 1,27 | 77,07 | 0,92 |
