# Supplementary material for: New Tools to Study DNA Double-Strand Break Repair Pathway Choice
Source: PLoS One. 2013 Oct 14;8(10):e77206. doi: 10.1371/journal.pone.0077206 (PMC3796453; doi:10.1371/journal.pone.0077206)
Supplement: Table S3 — Percentage of GFP and RFP expressing-cells from the BFP-positive pool in the SSR 2.0 system upon shRNA-mediated downregulation of NHEJ factors. (DOCX) [file pone.0077206.s004.docx]

**Table S3: Percentage of GFP and RFP expressing-cells from the BFP-positive pool in the SSR 2.0 system upon shRNA-mediated downregulation of NHEJ factors**

| shRNA | % RFP positive cells | | % GFP positive cells | | % GFP and RFP negative cells | |
| --- | --- | --- | --- | --- | --- | --- |
|  | **Average** | **SD** | **Average** | **SD** | **Average** | **SD** |
| Scramble | 10.25 | 1.41 | 17.08 | 2.36 | 72.67 | 3.77 |
| DNAPK | 16.75 | 0.71 | 11.75 | 1.18 | 71.50 | 1.89 |
| KU70 | 13.65 | 1.48 | 16.41 | 2.26 | 69.94 | 3.75 |
| KU80 | 14.7 | 1.48 | 17.23 | 0.40 | 68.07 | 1.08 |
| LIG4 | 13.275 | 1.27 | 17.47 | 4.24 | 69.25 | 2.97 |
