# Supplementary material for: New Tools to Study DNA Double-Strand Break Repair Pathway Choice
Source: PLoS One. 2013 Oct 14;8(10):e77206. doi: 10.1371/journal.pone.0077206 (PMC3796453; doi:10.1371/journal.pone.0077206)
Supplement: Table S4 — Percentage of GFP and RFP expressing-cells from the BFP-positive pool in the SSR 1.0 system upon shRNA-mediated downregulation of DNA resection. (DOCX) [file pone.0077206.s005.docx]

**Table S4: Percentage of GFP and RFP expressing-cells from the BFP-positive pool in the SSR 1.0 system upon shRNA-mediated downregulation of DNA resection.**

| shRNA | % RFP positive cells | | % GFP positive cells | | % GFP and RFP negative cells | |
| --- | --- | --- | --- | --- | --- | --- |
|  | **Average** | **SD** | **Average** | **SD** | **Average** | **SD** |
| Scramble | 8.52 | 1.05 | 14.44 | 1.78 | 77.05 | 2.83 |
| CtIP | 4.63 | 0.15 | 24.13 | 5.07 | 71.24 | 5.22 |
| Mre11 | 4.43 | 0.05 | 14.56 | 5.55 | 81.01 | 5.60 |
| BLM | 2.10 | 0.28 | 20.27 | 2.12 | 77.63 | 2.40 |
| Exo1 | 3.91 | 0.07 | 27.43 | 1.51 | 68.66 | 1.58 |
