# Supplementary material for: New Tools to Study DNA Double-Strand Break Repair Pathway Choice
Source: PLoS One. 2013 Oct 14;8(10):e77206. doi: 10.1371/journal.pone.0077206 (PMC3796453; doi:10.1371/journal.pone.0077206)
Supplement: Table S5 — Percentage of GFP and RFP expressing-cells from the BFP-positive pool in the SSR 2.0 system upon shRNA-mediated downregulation of DNA resection. (DOCX) [file pone.0077206.s006.docx]

**Table S5: Percentage of GFP and RFP expressing-cells from the BFP-positive pool in the SSR 2.0 system upon shRNA-mediated downregulation of DNA resection**

| shRNA | % RFP positive cells | | % GFP positive cells | | % GFP and RFP negative cells | |
| --- | --- | --- | --- | --- | --- | --- |
|  | **Average** | **SD** | **Average** | **SD** | **Average** | **SD** |
| Scramble | 7.27 | 0.07 | 11.72 | 0.11 | 81.01 | 0.18 |
| CtIP | 4.9 | 0.08 | 24.84 | 1.72 | 70.26 | 1.64 |
| Mre11 | 4.68 | 0.01 | 20.64 | 1.37 | 74.67 | 1.38 |
| BLM | 4.38 | 0.01 | 18.65 | 1.43 | 76.96 | 1.42 |
| Exo1 | 4.13 | 0.02 | 25.15 | 1.51 | 70.72 | 1.53 |
