# Supplementary material for: New Tools to Study DNA Double-Strand Break Repair Pathway Choice
Source: PLoS One. 2013 Oct 14;8(10):e77206. doi: 10.1371/journal.pone.0077206 (PMC3796453; doi:10.1371/journal.pone.0077206)
Supplement: Table S6 — Percentage of GFP and RFP expressing-cells from the BFP-positive pool in the SSR 1.0 system upon shRNA-mediated downregulation of the DNA damage response. (DOCX) [file pone.0077206.s007.docx]

**Table S6: Percentage of GFP and RFP expressing-cells from the BFP-positive pool in the SSR 1.0 system upon shRNA-mediated downregulation of the DNA damage response**

| shRNA | % RFP positive cells | | % GFP positive cells | | % GFP and RFP negative cells | |
| --- | --- | --- | --- | --- | --- | --- |
|  | **Average** | **SD** | **Average** | **SD** | **Average** | **SD** |
| Scramble | 8.60 | 0.28 | 12.15 | 0.40 | 79.25 | 0.68 |
| ATM | 4.59 | 0.10 | 14.62 | 5.30 | 80.79 | 5.40 |
| ATR | 3.77 | 0.11 | 36.55 | 1.35 | 59.68 | 1.46 |
| Brca1 | 3.38 | 0.05 | 13.04 | 2.83 | 83.58 | 2.88 |
| RNF168 | 2.63 | 1.15 | 15.82 | 0.61 | 81.56 | 1.76 |
| RNF8 | 2.84 | 1.02 | 23.31 | 2.89 | 73.85 | 1.87 |
| UBC13 | 2.49 | 0.84 | 33.80 | 5.91 | 63.71 | 6.75 |
| Pias1 | 3.46 | 1.03 | 26.30 | 5.35 | 70.24 | 6.38 |
| Pias4 | 4.24 | 0.06 | 16.19 | 7.54 | 79.57 | 7.61 |
| UBC9 | 1.95 | 0.32 | 26.48 | 4.62 | 71.57 | 4.93 |
