# Supplementary material for: New Tools to Study DNA Double-Strand Break Repair Pathway Choice
Source: PLoS One. 2013 Oct 14;8(10):e77206. doi: 10.1371/journal.pone.0077206 (PMC3796453; doi:10.1371/journal.pone.0077206)
Supplement: Table S7 — Percentage of GFP and RFP expressing-cells from the BFP-positive pool in the SSR 2.0 system upon shRNA-mediated downregulation of the DNA damage response. (DOCX) [file pone.0077206.s008.docx]

**Table S7: Percentage of GFP and RFP expressing-cells from the BFP-positive pool in the SSR 2.0 system upon shRNA-mediated downregulation of the DNA damage response**

| shRNA | % RFP positive cells | | % GFP positive cells | | % GFP and RFP negative cells | |
| --- | --- | --- | --- | --- | --- | --- |
|  | **Average** | **SD** | **Average** | **SD** | **Average** | **SD** |
| Scramble | 7.58 | 0.29 | 13.69 | 3.09 | 78.72 | 2.80 |
| ATM | 4.84 | 0.04 | 19.07 | 2.16 | 76.09 | 2.12 |
| ATR | 3.99 | 0.06 | 20.14 | 1.51 | 75.88 | 1.45 |
| Brca1 | 3.56 | 0.01 | 23.95 | 2.39 | 72.48 | 2.38 |
| RNF168 | 4.62 | 0.02 | 24.40 | 1.73 | 70.98 | 1.70 |
| RNF8 | 4.33 | 0.17 | 23.92 | 5.93 | 71.75 | 6.10 |
| UBC13 | 4.01 | 0.04 | 21.72 | 1.15 | 74.27 | 1.11 |
| Pias1 | 5.31 | 0.01 | 18.27 | 1.87 | 76.42 | 1.86 |
| Pias4 | 4.49 | 0.01 | 21.75 | 1.45 | 73.75 | 1.45 |
| UBC9 | 3.43 | 0.12 | 23.58 | 5.74 | 72.98 | 5.86 |
